# Intraoperative Neurophysiological Monitoring in Spina Bifida and Tethered Cord Surgeries: A Systematic Review of Clinical Outcomes

**DOI:** 10.7759/cureus.115612

**Published:** 2026-09-01

**Authors:** Vizmary J Montes-Peña, Ana M Ozguc, Pravallika Srusti, Rutaba Ali, Swarna Pashine, Faisal R Jahangiri

**Affiliations:** 1 Department of Neurology, Division of Clinical Neurophysiology, King Abdulaziz Medical City, Riyadh, SAU; 2 Neurology, King Abdullah Specialized Children’s Hospital, Ministry of National Guard Health Affairs, Riyadh, SAU; 3 Department of Neuroscience, School of Behavioral and Brain Sciences, University of Texas at Dallas, Richardson, USA; 4 Clinical and Surgical Neurophysiology, Global Innervation LLC, Dallas, USA

**Keywords:** electromyography, intraoperative neuromonitoring, neuromonitoring, spina bifida, surgical outcomes, tethered cord syndrome

## Abstract

Spina bifida and tethered cord syndrome (TCS) are forms of spinal dysraphism requiring surgical intervention to prevent progressive neurological decline. Intraoperative neurophysiological monitoring (IONM) provides real-time feedback, allowing surgeons to minimize intraoperative injury during these procedures. This systematic review analyzed 19 primary studies published between 2015 and 2025 examining the role of IONM techniques, including somatosensory evoked potentials (SSEP), motor evoked potentials (MEP), electromyography (EMG), and bulbocavernosus reflex (BCR), in spina bifida and tethered cord surgeries. Methodological quality and risk of bias were formally appraised using the Methodological Index for Non-Randomized Studies (MINORS) criteria. Multimodal IONM use was associated with favorable postoperative motor outcomes. Conversely, bladder and sphincter outcomes were more variable, particularly in complex or reoperative cases. The included literature demonstrated an overall moderate risk of bias, driven primarily by retrospective designs and lack of blinded outcome assessments. Longer-term comparative data suggested that differences between monitored and unmonitored cohorts may become more apparent over time, although this pattern remains hypothesis-generating. In lower-risk surgeries such as isolated filum terminale release, patients achieved favorable outcomes regardless of monitoring status, indicating limited added value of IONM in this subgroup. These findings support the selective, risk-adapted use of IONM while highlighting a moderate baseline risk of bias in the current literature. This underscores the need for standardized protocols and high-quality prospective studies with extended follow-up.

## Introduction and background

Congenital spinal pathologies present unique challenges for surgical management because neural structures may be malformed, displaced, tethered, or intimately associated with abnormal tissues. Surgical correction aims not only to address the underlying anatomical abnormality but, critically, to preserve existing neurological function and prevent new postoperative deficits. In patients with spinal dysraphism, the distinction between functional neural tissue and structures that can be safely manipulated or divided may be difficult to determine based on anatomy alone. This is particularly important in procedures involving tethered cord release, myelomeningocele repair, lipomyelomeningocele, filum terminale abnormalities, and other congenital lesions in which motor, sensory, and sacral neural pathways may be at risk during surgical dissection.

Intraoperative neurophysiological monitoring (IONM) provides real-time functional assessment of the nervous system during these procedures and has therefore become an important adjunct to the surgical management of congenital spinal pathology. Unlike anatomical visualization alone, IONM allows the surgical team to assess the functional integrity of neural pathways, identify neural structures, detect potentially harmful changes during surgical manipulation, and guide corrective interventions before neurological injury becomes irreversible. These functions are especially relevant in pediatric patients, in whom preoperative neurological examination may be limited and congenital anatomical abnormalities can make functional localization particularly challenging.

Spinal dysraphism encompasses a heterogeneous group of congenital abnormalities resulting from altered development of the neural tube and surrounding structures. These include open lesions, such as myelomeningocele, as well as closed forms including spina bifida occulta, lipomyelomeningocele, and filum terminale lipomas [1]. Depending on the type and severity of the lesion, affected children may develop motor weakness, sensory impairment, scoliosis, gait abnormalities, and bladder or bowel dysfunction. Surgical intervention may be required to repair the congenital defect, release a tethered spinal cord, preserve neurological function, or prevent progressive neurological deterioration. In selected cases, repair may be performed prenatally, although postnatal surgery remains necessary for many patients [2].

The complex and variable neuroanatomy encountered during these operations provides a strong rationale for multimodality IONM. Somatosensory evoked potentials (SSEPs) assess ascending sensory pathway integrity, while transcranial motor evoked potentials (MEPs) provide information regarding descending motor pathways. Free-running and triggered electromyography (EMG) can detect mechanical irritation of motor roots and help distinguish functional neural tissue from non-neural structures [3]. The bulbocavernosus reflex (BCR) provides additional assessment of sacral reflex pathways and may be particularly relevant during tethered cord and other lumbosacral procedures where preservation of bowel, bladder, and sexual function is an important surgical objective. Train-of-four (TOF) peripheral nerve stimulation has also been used adjunctively in some studies to assess the degree of neuromuscular blockade and ensure that residual pharmacological paralysis does not compromise interpretation of EMG and MEP responses; however, TOF should be distinguished from the neurophysiological modalities used directly to monitor neural function.

Beyond conventional SSEPs, transcranial muscle motor evoked potentials (mMEPs), and EMG, direct wave (D-wave) monitoring plays a critical role in complex or reoperative spinal dysraphism surgeries. Unlike mMEPs, which are recorded from peripheral target muscle groups and remain highly sensitive to volatile anesthetic suppression, the D-wave is captured directly from the spinal cord surface using an epidural or subdural electrode array placed proximal and distal to the surgical site. This modality monitors the descending corticospinal tract seamlessly, providing continuous data uninhibited by neuromuscular blockade. D-wave monitoring plays a crucial role during the resection of split cord malformations (SCMs) and diastematomyelia, in which longitudinal division of the spinal cord creates highly distorted structural vectors. During the delicate removal of midline bony or fibrous septa, maintaining a stable D-wave amplitude provides the surgical team with a reliable, predictable indicator of motor tract preservation, allowing surgery to proceed safely even when peripheral muscle responses experience transient, baseline fluctuations.

Despite the increasing use of IONM in congenital spinal surgery, its application remains nonuniform, and uncertainty persists regarding which modalities provide the greatest clinical value, whether multimodality monitoring improves neurological outcomes compared with surgery without IONM, and how monitoring strategies should be tailored to specific congenital pathologies and surgical procedures. A clearer understanding of the available evidence is therefore important for defining the role of IONM in protecting neurological function during these technically demanding operations.

Accordingly, the objectives of this paper are to (1) review the current applications of IONM in surgery for congenital spinal dysraphism, including spina bifida and tethered cord syndrome (TCS); (2) compare neurological outcomes between procedures performed with and without IONM; and (3) evaluate the relative utility of individual and multimodal neurophysiological monitoring techniques for identifying neural structures and preserving motor, sensory, and sacral neurological function.

## Review

Methods: search strategy and study selection

Information Sources

Literature searches were conducted across PubMed, supplemented by manual, citation-based tracking of secondary sources. The final search architecture was completed in February 2026 [4].

Search Architecture

A two-branched Boolean approach was deployed on PubMed:

Branch 1 (Somatic/IONM Focus): "spina bifida neuromonitoring," "spina bifida ionm," "spina bifida neuromonitoring ionm," "spina bifida intraoperative neuromonitoring," "myelomeningocele ionm," "myelomeningocele neuromonitoring," "myelomeningocele intraoperative neuromonitoring," and "myelomeningocele ionm neuromonitoring."

Branch 2 (Anatomical/Detethering Focus): "tethered cord syndrome intraoperative neuromonitoring," "tethered cord ionm," "tethered cord neuromonitoring," "detethering neuromonitoring," "detethering ionm," "untethering neuromonitoring," "untethering ionm," and "spinal cord detethering intraoperative neuromonitoring."

Eligibility Criteria

Inclusion was strictly restricted to peer-reviewed, English-language clinical studies published between 2015 and 2025. Studies were required to report a direct correlation between IONM findings and postoperative clinical outcomes (neurological, urological, orthopedic, pain, or functional).

Quality and Risk-of-Bias Appraisal

Methodological quality was evaluated using the Methodological Index for Non-Randomized Studies (MINORS) criteria. Non-comparative cohorts were evaluated on eight items (maximum score: 16). Comparative cohorts were evaluated across 12 items (maximum score: 24).

Screening and Selection Flow

Initial database yield: Branch 1 yielded 31 unique records; Branch 2 yielded 70 unique records.

Deduplication: Removal of overlapping records between the two primary PubMed branches eliminated 26 duplicates, resulting in 75 unique database records.

Supplementary identification: Manual citation-tracking identified six additional studies. One supplementary eligible paper [3], missed by the keyword strings, was manually added, bringing the total identification yield to 82 records.

Title and abstract screening: Fifty-four records were excluded during initial title and abstract screening due to a lack of relevance to IONM use in spina bifida or tethered cord surgeries.

Full-text eligibility assessment: Twenty-eight full-text reports were sought and successfully retrieved for comprehensive eligibility analysis.

Exclusion Analysis

Ten reports were excluded during full-text evaluation for the following explicit structural reasons: book chapters without original research (*n = 1*), narrative reviews lacking raw clinical patient data (*n = 2*), preprints/systematic reviews retained strictly for background context (*n = 1*), technical feasibility papers missing postoperative outcome correlations (*n = 3*), duplicate datasets captured by larger included cohorts (*n = 1*), studies omitting intraoperative neuromonitoring entirely (*n = 1*), and surgical technique case series lacking outcome data (*n = 1*)

Final Synthesis Selection

The evaluation yielded 19 studies that met all definitive inclusion criteria for qualitative synthesis (Figure 1; Tables 1-2).

**Figure 1:**
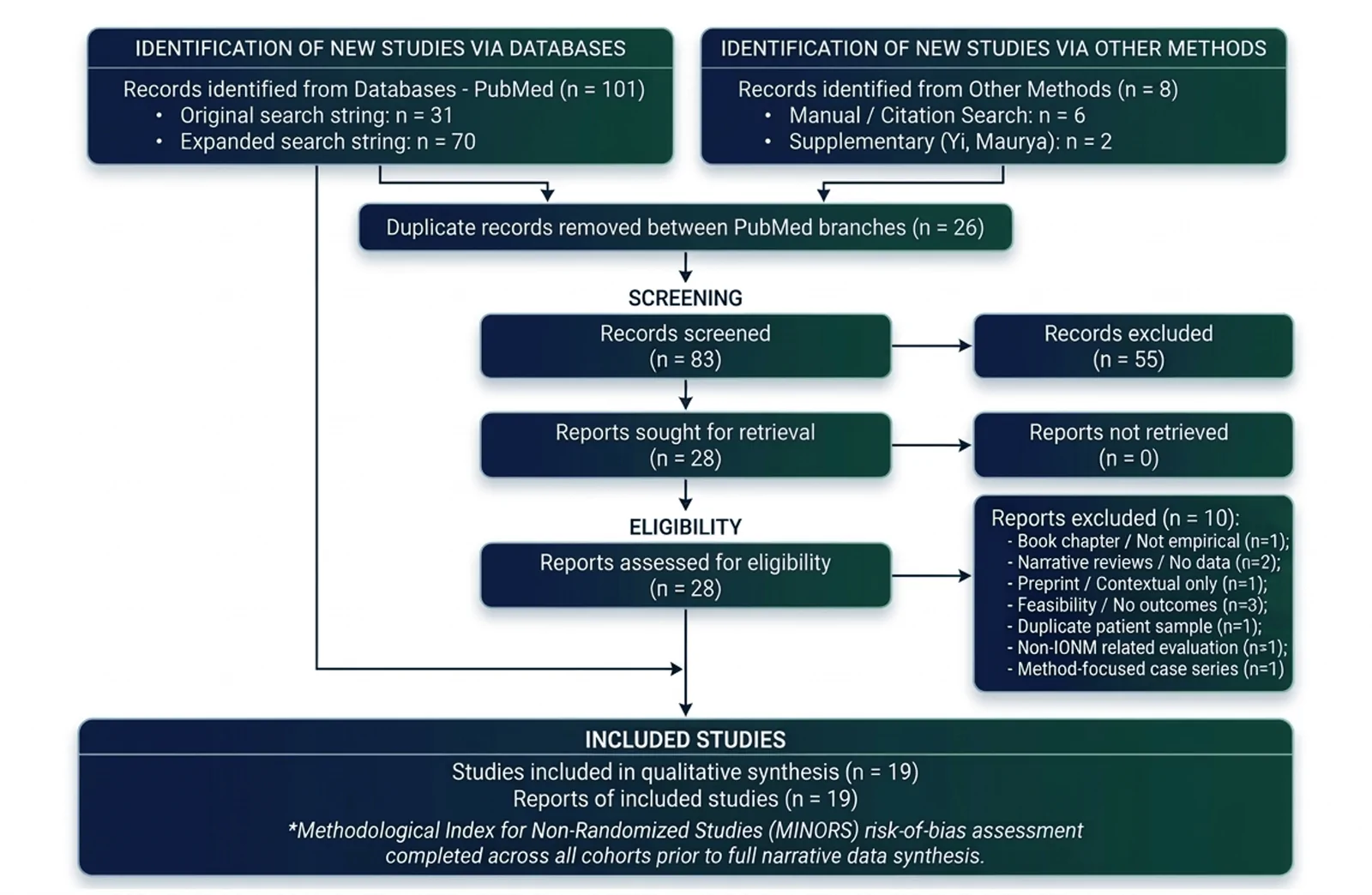
PRISMA flow diagram of study identification, selection, and appraisal. PRISMA 2020 flow diagram illustrating the systematic study selection process. Initial database queries across two PubMed search branches yielded an original search (*n = 31*) and an expanded tethered cord-related search (*n = 70*), resulting in 75 unique records after the removal of 26 duplicates between branches. Seven additional records were identified via secondary methods, including manual citation-tracking (*n = 6*) and one supplementary keyword-omitted record, producing a total pool of 82 identified records. Following title and abstract screening, 54 irrelevant records were excluded. Of the 28 full-text reports successfully retrieved and assessed for eligibility, 10 were excluded based on strict structural criteria: book chapters without original data (*n = 1*), narrative reviews (*n = 2*), preprints/systematic reviews retained only for background context (*n = 1*), technical feasibility studies lacking clinical outcome correlation (*n = 3*), duplicate patient populations (*n = 1*), absence of intraoperative neuromonitoring (IONM) data (*n = 1*), and technique-focused case series without clinical correlation (*n = 1*). The final 19 eligible studies underwent formal methodological quality and risk-of-bias appraisal using the Methodological Index for Non-Randomized Studies (MINORS) criteria prior to qualitative synthesis and final inclusion.

**Table 1:** Characteristics of the included studies. Anesthesia and IONM technique details are reported as “Not specified” where the original source did not describe them; this reflects incomplete reporting in the primary literature rather than incomplete extraction. TIVA, total intravenous anesthesia; TcMEP, transcranial motor evoked potential; SSEP, somatosensory evoked potential; EMG, electromyography (free-run and/or triggered); BCR, bulbocavernosus reflex; CUSA, Cavitron ultrasonic aspirator; MMC, myelomeningocele; TCS, tethered cord syndrome

Study	N	Age range	Anesthesia	IONM techniques	Diagnosis	Risk-of-bias assessment (MINORS score)
Flanders et al., 2023 [2]	51	Pediatric (avg. 2y 10mo)	TIVA	TcMEP, SSEP, EMG, EEG	Tethered cord (after fetal MMC closure)	11/16 (Moderate risk; retrospective data)
Yi et al., 2019 [3]	25 (19 TCS/SB-related)	Pediatric (0–3 mo)	TIVA	TcMEP, SSEP, BCR	Tethered cord	10/16 (Moderate risk; small sample size)
Finger et al., 2020 [5]	32 pts / 38 procedures	Adults	Not specified	Free-running EMG, MEP, BCR	Secondary tethered cord syndrome (adults)	11/16 (Moderate risk; retrospective data)
Dulfer et al., 2017 [6]	65 (60 reassessed)	Mixed pediatric/adult	Not specified	Mapping (direct nerve root stimulation) + monitoring (TcMEP)	Tethered cord (lipomyelomeningocele, SCM, MMC, tight filum)	12/16 (Low-to-moderate risk; excellent long-term follow-up)
Siller et al., 2020 [7]	12	8 pediatric, 4 adult (< 33y)	TIVA	TcMEP, SSEP, EMG	Intraspinal epidermoid/dermoid cysts	9/16 (Moderate-to-high risk; very small sample size)
Gadhvi et al., 2023 [8]	26	1–56 y	Not specified	t-EMG, TcMEP, f-EMG	Tethered cord syndrome	9/16 (Moderate risk; missing follow-up endpoints)
Udayakumaran et al., 2024 [9]	208	Pediatric < 15y (mean 3.75y)	TIVA (fentanyl-propofol/dexmedetomidine)	TcMEP, direct stimulation/mapping	Tethered cord syndrome (multiple dysraphic pathologies)	13/16 (Low risk; large, well-characterized cohort)
McGrath et al., 2024 [10]	21	Pediatric (2–17y)	Not specified	TcMEP, t-EMG	Tethered cord	10/16 (Moderate risk; single-center retrospective)
Lalgudi Srinivasan et al., 2021 [11]	80 (37 w/ IONM, 43 w/o IONM)	Pediatric (75% < 3y)	TIVA	TcMEP, SSEP, EMG	Filum terminale lipoma	18/24 (Low-to-moderate risk; comparative design used)
Krause et al., 2025 [12]	7	Newborn infants	TIVA	MEP, SSEP, EMG, BCR	Open neural tube defect (postnatal MMC repair)	9/16 (Moderate-to-high risk; very small newborn cohort)
McDevitt et al., 2025 [13]	122	Pediatric (median 3y, IQR 2–9)	TIVA	MEP, SSEP, EMG, BCR	Tethered cord	12/16 (Low risk; robust multi-year pediatric cohort)
Fekete et al., 2019 [14]	91 pts / 102 surgeries	Pediatric (mostly)	Not specified	EMG (direct/triggered stimulation); BCR in 8 cases, later abandoned	Tethered cord syndrome	11/16 (Moderate risk; historical cohort issues)
Guo et al., 2024 [15]	454	Pediatric	Not specified	Tc-MEP, SSEP, free-running EMG, triggered EMG	Tethered cord syndrome	13/16 (Low risk; very high power / large sample size)
Jiang et al., 2020 [16]	100	Pediatric	Not specified	EMG with nerve stimulator; CUSA used as resection tool (not as mapping device)	Tethered cord syndrome	11/16 (Moderate risk; single center series)
Squintani et al., 2025 [17]	50 surgeries	Not specified	Not specified	TcMEP, tibial SSEP (TNSEP), pudendal-anal reflex (PAR)	Tethered cord syndrome	10/16 (Moderate risk; missing explicit age demographics)
Flanders et al., 2020 [18]	1	Newborn (15 days old)	TIVA	MEP, SSEP, EMG, BCR	Thoracic myelocystocele	7/16 (High risk; single-patient case report protocol)
Ezgu et al., 2020 [19]	8	Newborns	Not specified	Free-running EMG, direct stimulation of neural placode/nerve roots	Myelomeningocele (thoracolumbar/lumbosacral)	9/16 (Moderate-to-high risk; small newborn cohort)
Eibach et al., 2021 [20]	8	Newborn infants	Not specified	TcMEP, SSEP, EMG, BCR	Open neural tube defect	9/16 (Moderate-to-high risk; small infant cohort
Maurya et al., 2016 [21]	22	Mixed pediatric & adult (Mean 13.8y)	Not specified	SSEP	Tethered cord syndrome	13/16 (Low risk; robust prospective protocol)

**Table 2:** Study design, follow-up, and key findings of the included studies. Evidence levels reflect study design only (Level III, retrospective comparative cohort; Level IV, retrospective or prospective case series without a non-IONM comparator; Level V, single case report) and were assigned by the review authors based solely on design. No formal risk-of-bias instrument was applied, consistent with the narrative rather than systematic nature of this review; these levels should therefore be read as a design-based summary rather than a formal quality grading. Methodological quality was calculated using the MINORS framework: items were scored as 0 (not reported), 1 (reported but inadequate), or 2 (reported and adequate). Comparative studies are evaluated out of a global ideal score of 24; non-comparative studies out of 16. SCM, split cord malformation; FTL, filum terminale lipoma; TNSEP, tibial nerve somatosensory evoked potential; PAR, pudendal-anal reflex (functionally equivalent to the bulbocavernosus reflex). “Comparator” indicates whether the study included a non-IONM or historical comparison group.

Study	Design (comparator)	Follow-up	Principal finding	Limitations / evidence level
Flanders et al., 2023 [2]	Retrospective cohort, no comparator	~4 months postop	Electrophysiologic functional level 2.5 levels better than anatomic level; 90% unreliable/undetectable lower-extremity SSEP	No control group, single center; Level IV Evidence (Moderate Risk of Bias)
Yi et al., 2019 [3]	Retrospective case series, no comparator	Immediate postop	TcMEP recorded in 24/25 infants; feasible < 3 mo of age	Small sample size, no control arm; Level IV Evidence (Moderate Risk of Bias)
Finger et al., 2020 [5]	Retrospective cohort, no comparator	Mean 48 months	IONM associated with improved weakness/paresthesia; 16% retethering rate	Adults only, no comparative arm; Level IV Evidence (Moderate Risk of Bias)
Dulfer et al., 2017 [6]	Retrospective, no comparator	Mean 11.2 y	Neurological, urological, and pain domains stable or improved long-term; progressive scoliosis in a subset	Potential historical recall bias; Level IV Evidence (Low-to-Moderate Risk)
Siller et al., 2020 [7]	Retrospective case series, no comparator	Median 4.8 y	Gross total resection 92%; no postoperative neurological deterioration	Very small cohort (n=12), single center; Level IV Evidence (Moderate-to-High Risk)
Gadhvi et al., 2023 [8]	Retrospective case series, no comparator	Not specified	No new postoperative deficit; TcMEP stable throughout surgery	Unspecified follow-up length, high dropout risk; Level IV Evidence (Moderate Risk)
Udayakumaran et al., 2024 [9]	Prospective retrospective, no comparator	1 y	Motor/sphincter monitorability 99.4%/89.3%; 91% of patients with motor deficit improved	Retrospective design, heterogeneous dysraphic pathologies included; Level IV Evidence (Low Risk of Bias)
McGrath et al., 2024 [10]	Retrospective review, no comparator	Not specified	TcMEP is below clinical function level in 95.2% of cases	Small sample size, single-center retrospective dataset; Level IV Evidence (Moderate Risk of Bias)
Lalgudi Srinivasan et al., 2021 [11]	Retrospective, comparative (IONM vs. no IONM)	Mean 49.1 months	No neurological morbidity in either group; IONM not associated with added benefit in FTL untethering	Non-randomized allocation, single-center retrospective comparative cohort; Level III Evidence (Low-to-Moderate Risk of Bias)
Krause et al., 2025 [12]	Retrospective case series, no comparator	Up to 7 y	85% showed ≥ 1-level motor gain vs. anatomical level	Very small newborn sample size, lack of an unmonitored comparison arm; Level IV Evidence (Moderate-to-High Risk of Bias)
McDevitt et al., 2025 [13]	Retrospective cohort, no comparator	3 months	Modality success 100/99/90/73% (EMG/TcMEP/SSEP/BCR); 11% alert breaches, 85% resolved without permanent deficit	Retrospective review, lack of randomized controls; Level IV Evidence (Low Risk of Bias)
Fekete et al., 2019 [14]	Retrospective, comparative (different eras)	Subset to long-term (69 pts)	Perioperative risk reduced 9.4%→2.9% with IONM; long-term progression avoided in 88.7%	Historical cohort changes, high rate of abandoned modalities; Level IV Evidence (Moderate Risk of Bias)
Guo et al., 2024 [15]	Retrospective, comparative	Short + medium/long-term	No significant short-term benefit; significant medium- to long-term benefit with IONM	Retrospective nature, single-institution data despite high power; Level IV Evidence (Low Risk of Bias)
Jiang et al., 2020 [16]	Retrospective, comparative (2 eras)	Not specified	Total release 80% vs. 71%; new complications 18% vs. 37% (monitored vs. unmonitored)	Confounding surgical variables (CUSA tool use), no external control group; Level IV Evidence (Moderate Risk of Bias)
Squintani et al., 2025 [17]	Retrospective case series, no comparator	Not specified	High sensitivity/specificity for TcMEP and TNSEP; PAR showed low sensitivity, no significant outcome correlation	Incomplete demographic reporting, retrospective review; Level IV Evidence (Moderate Risk of Bias)
Flanders et al., 2020 [18]	Case report	25 months	Feasibility of IONM demonstrated at 15 days of life	Single-patient protocol, findings lack statistical generalizability; Level V Evidence (High Risk of Bias)
Ezgu et al., 2020 [19]	Retrospective case series, no comparator	Not specified	Nerve root stimulation responses are more robust than placode stimulation responses	Small newborn cohort size, short-term follow-up window; Level IV Evidence (Moderate-to-High Risk of Bias)
Eibach et al., 2021 [20]	Prospective case series, no comparator	Mean 24 months	No postoperative neurological worsening; no inclusion of dermoid cysts with new resection paradigm	Small infant population size, no comparative control cohort; Level IV Evidence (Moderate-to-High Risk of Bias)
Maurya et al., 2016 [21]	Prospective cohort, no comparator	3 months postop	Surgical detethering significantly improves SSEP latencies and reduces biochemical markers of active neuronal injury (NSE/S100B).	Short follow-up window; lacks an unmonitored control group; Level IV Evidence (Low Risk of Bias)

Patients ranged from newborns to adults. One study enrolled adults exclusively [5]. Four additional studies included mixed pediatric and adult cohorts [6-8]. Where the anesthetic technique was reported, total intravenous anesthesia (TIVA) with propofol and fentanyl or remifentanil was the most commonly described approach; anesthetic protocol was not specified in several of the more recently identified studies. Data collection consisted primarily of retrospective or prospective chart review. TcMEP and EMG were the most frequently reported modalities, while SSEP and BCR were reported less consistently; several studies, including one of the largest cohorts, explicitly excluded BCR from their protocol [9]. Direct nerve root mapping was reported in individual studies. Use of an ultrasonic surgical aspirator was noted in one included study as an adjunct during lipoma resection rather than as an independent mapping modality. Study characteristics are summarized in Table 1.

Intraoperative Neurophysiological Monitoring

TcMEP and EMG were the most consistently reported modalities across the included studies, while SSEP and BCR were used more variably; the specific combination and level of technical detail reported varied substantially across studies. Where reported, EMG needle electrodes were most commonly placed in the lower extremities, including the bilateral quadriceps, hamstrings, tibialis anterior, gastrocnemius, abductor hallucis, and external anal sphincter. For SSEP, the posterior tibial nerve was the most commonly stimulated site bilaterally, with cortical responses recorded via scalp electrodes following the international 10-20 system. Upper-extremity SSEP, most often via median or ulnar nerve stimulation, was reported less consistently; where used, it served either as an intraoperative control to help distinguish an anesthetic or technical cause of amplitude change from a genuine neurological event, or, in prone positioning, to monitor brachial plexus integrity. For BCR, the afferent arc was stimulated at the dorsal penile or clitoral nerve, with the reflex response recorded from the external anal sphincter and, in some protocols, the bulbocavernosus muscle directly. Alert and stimulation criteria were not consistently reported across studies.

Results

Across the included studies, IONM was used to support intraoperative decision-making and detect potential neural compromise. Comparative observational cohorts reported fewer postoperative complications in selected settings, although causal inference was limited by retrospective design and temporal confounding.

Multimodal protocols incorporating MEP, SSEP, EMG, and BCR were used to detect neural irritation or functional change during delicate dissection. Transcranial MEPs were recorded in all 21 reoperations after fetal myelomeningocele repair in one series, identifying functioning nerve roots below the lesion level; 52% of patients had MEPs detected two spinal levels below their expected function, and EMG findings matched MEP findings in 85% of cases [10]. In a related series of 51 patients undergoing tethered cord release after prior open fetal myelomeningocele closure, the electrophysiologic functional level averaged 2.5 levels better than the anatomical level, and manual motor testing remained largely unchanged postoperatively; however, 90% of patients had unreliable or undetectable lower-extremity SSEPs, revealing a consistent motor-sensory imbalance in this population [2].

In filum terminale lipoma (FTL) surgeries, IONM utility was more limited. Eighty children undergoing FTL untethering, 37 with IONM and 43 without, showed no neurological morbidity in either group and stable long-term outcomes in one series [11]. The authors concluded that IONM is not necessary for most FTL cases in experienced hands, though it helped confirm the absence of neural structures when anatomy was unclear. In a series of 7 newborns undergoing postnatal myelomeningocele repair, IONM provided clear EMG, MEP, and BCR signals from the first days of life [12]. Two patients showed early postoperative neurological decline, but 85% had improved motor outcomes relative to their predicted lesion level by follow-up.

One of the largest cohorts with detailed neurophysiological outcome data in this review followed 208 consecutive children who underwent detethering for TCS under IONM with TcMEP and direct mapping over a nine-year period [9]. Motor and sphincter monitorability reached 99.4% and 89.3%, respectively; sensitivity for predicting new neurological deficit was 76.1%, and specificity was 98.3%. At one-year follow-up, 91% of patients with motor deficits, 82.3% with bladder deficits, and 88.8% with bowel deficits showed improvement. Among a subgroup of 87 infants in this cohort, monitorability and diagnostic accuracy were comparable to those of the wider group, indicating that IONM remains feasible even in the youngest patients. In another large series, 122 children undergoing tethered cord untethering with combined IONM and mapping had successful EMG, MEP, SSEP, and BCR recordings in 100%, 99%, 90%, and 73% of cases, respectively; alert criteria breaches occurred in 11% of patients, and 85% of these resolved without permanent deficit [13].

Long-term outcomes after IONM-assisted detethering were addressed directly in a study that reassessed 60 of 65 patients originally reported, with a mean follow-up of 11.2 years [6]. Neurological, urological, and pain domains remained stable or slightly improved over the long term. Because this cohort was monitored throughout without a non-IONM comparator, these findings describe durable stability after IONM-assisted detethering rather than demonstrate that IONM itself caused this stability. Progressive scoliosis was noted in a subset of patients, particularly young females, indicating that detethering does not fully prevent orthopedic progression, regardless of IONM use.

Comparative cohort studies suggested an association between IONM and improved long-term outcomes, although the non-monitored surgeries were performed in an earlier era, leaving temporal confounding unresolved. In a comparison of 91 patients undergoing 102 tethered cord surgeries, perioperative surgical risk decreased from 9.4% without electrophysiological monitoring to 2.9% with it, and long-term neurological progression was avoided in 88.7% of patients with electrophysiological monitoring [14]. In a larger series of 454 children, short-term outcomes did not differ significantly between monitored and unmonitored groups, but medium- to long-term follow-up showed a significantly better response rate (32.1% versus 23.7%) and lower deterioration rate (3.3% versus 8.5%) in the IONM group [15]. A retrospective comparison of 100 children similarly found that IONM was associated with more complete surgical release, fewer new complications (18% versus 37%), and higher rates of complete recovery or significant improvement (76% versus 54%) [16].

In adults, secondary TCS was evaluated in 32 patients undergoing 38 untethering procedures, with a 16% retethering rate; IONM was associated with a positive effect on postoperative weakness and paresthesia [5]. In a smaller series of 26 patients aged 1 to 56 years, monitored with t-EMG, TcMEP, and f-EMG, successful identification and detethering of nerve roots and the filum terminale were reported in all cases, with no new postoperative deficits [8]. In a series of 12 patients undergoing resection of intraspinal epidermoid or dermoid cysts under multimodal IONM (EMG, SSEP, TcMEP, and pudendal/anal sphincter monitoring), gross total resection was achieved in 92% of cases with no postoperative neurological deterioration in any patient, spanning both pediatric and adult cohorts [7]. A series of 50 TCS surgeries monitored with TcMEP, tibial SSEP, and the pudendal-anal reflex evaluated predictive values for postoperative outcome, finding high sensitivity and specificity for TcMEP and tibial SSEP changes with significant correlation to clinical outcome, whereas the pudendal-anal reflex showed low sensitivity and no significant correlation with urinary or anal function [17].

MEPs were successfully recorded in 24 of 25 infants under 3 months of age during TIVA in another series; the single patient with long-term weakness showed a significant intraoperative decrease in MEP amplitude [3]. IONM feasibility has been demonstrated even in the immediate neonatal period, with successful MEP, SSEP, and BCR recordings reported in an infant as young as 15 days of life undergoing thoracic myelocystocele resection [18]. Free-running EMG and direct stimulation of the neural placode and nerve roots were used in 8 newborns undergoing MMC repair in another study [19]. Nerve root stimulation produced more robust and consistent responses than placode stimulation, and only 1 of 8 patients showed no lower-extremity response, supporting the role of IONM in identifying residual functional neural tissue within the placode during reconstruction. In a separate series of 8 newborns undergoing postnatal repair of open neural tube defects, resection of the non-functional portion of the neural placode guided by direct stimulation and IONM resulted in no postoperative neurological worsening and no inclusion of dermoid cysts [20].

SSEPs were harder to interpret in young patients due to immature sensory pathways and anesthesia-related variability. Therefore, neurodevelopmental maturity and anesthesia noise require careful handling. False positives from filum stimulation occasionally prolonged surgery without causing lasting harm. The lack of standardized signal thresholds and inconsistent use of modalities across institutions limited direct comparisons between studies. Addressing heterogeneous signal thresholds will improve future meta-analyses.

Discussion

Spina bifida is among the most severe neural tube defects requiring complex surgical intervention. IONM provides real-time feedback on neural function to prevent surgical complications during these procedures. IONM use in spina bifida and TCS surgery has increased, although implementation remains inconsistent, as its clinical value depends on the surgical case.

For contextual comparison, a separate systematic review of aggregate data reported that across 1,492 surgeries from the broader published literature, IONM use was associated with a lower rate of postoperative neurological deficits: 7.25% with IONM versus over 15% without [22]. This figure is presented here as background context from outside the primary studies synthesized in this review rather than as a direct finding of the included cohorts.

Postnatal myelomeningocele repair in newborns monitored with MEP, SSEP, EMG, and BCR showed that IONM identified mechanical stress on neural structures and guided nerve root preservation, with 85% of children showing improved motor function at two-year follow-up [12]. MEP monitoring has similarly been shown to be safe and effective in infants under three months of age [3]. Direct placode and nerve root stimulation has similarly demonstrated preserved functional tissue within the neural placode during myelomeningocele repair [19]. A similarly designed neonatal series supported the feasibility of a more aggressive, monitoring-guided resection paradigm for open neural tube defects [20]. The 208-patient cohort reported by Udayakumaran et al. further supports the diagnostic value of IONM in tethered cord surgery, with high sensitivity and specificity in predicting motor deficits [9]. A large single-center series combining IONM with direct mapping corroborated these high success rates across all four modalities [13]. Data from patients undergoing tethered cord release after prior fetal myelomeningocele closure similarly showed preserved motor function despite frequently unreliable sensory potentials, a pattern that further supports the applicability of IONM across a wide range of surgical contexts [2].

A pattern that emerged consistently across the newly incorporated tethered cord literature is that the measurable benefit of IONM tends to widen with longer follow-up. Short-term comparisons between monitored and unmonitored cohorts showed little or no significant difference in one large series, whereas medium- to long-term follow-up in the same cohort revealed a significantly higher response rate and a lower deterioration rate in the monitored group [15]. Independently, an 11.2-year reassessment of an IONM-monitored cohort found lasting stability in neurological, urological, and pain domains [6]. Together, these findings suggest that studies relying solely on short-term postoperative assessments may underestimate medium- to long-term differences between monitored and unmonitored cohorts. Given the limited number of studies with both an adequate comparator and long-term follow-up, this pattern should be considered hypothesis-generating rather than established.

The need for IONM in lower-risk scenarios has been challenged by findings that 80 pediatric patients with filum terminale lipomas (FTLs) had no neurological deficits after untethering regardless of IONM use [11]. The authors concluded that, while reassuring intraoperatively, IONM is unnecessary for FTL given the procedure's low neurological risk.

Findings in the adult population, historically underrepresented in this literature, were consistent with the pediatric data. Secondary TCS in adults showed a meaningful retethering rate and a positive association between IONM and improvement in weakness and paresthesia [5]. Long-term follow-up of a mixed pediatric-adult cohort supported durable neurological and urological stability after IONM-assisted detethering [6]. These findings extend the applicability of IONM-based surgical decision-making beyond the pediatric population that has dominated prior literature in this field.

Several gaps remain despite growing support for IONM. Many favorable-outcome studies have small sample sizes, limiting external validity, and protocols are not standardized across institutions: some centers use a full MEP, SSEP, EMG, and BCR suite, while others use one or two modalities. Cost-effectiveness across surgical contexts remains largely unstudied, a gap most relevant to resource-limited settings where financial and logistical constraints affect implementation. IONM may not change surgical management in low-risk procedures such as FTL untethering [11], but omitting it in complex or high-risk cases could allow avoidable nerve damage. This gap creates uncertainty in clinical decision-making in pediatric neurosurgery, where preserving long-term function is the primary goal.

The IONM rationale aligns with the two-hit hypothesis [12]: a first developmental hit produces spinal cord malformation, and a second intraoperative hit risks further neural injury during surgical correction. IONM serves as a neuroprotective tool that reduces the risk of the second hit by enabling surgeons to respond to changes in neural signal integrity, supporting its use in complex or high-risk cases where neural elements are difficult to identify.

Improved postoperative motor outcomes in pediatric patients have been linked to IONM use, countering concerns that signal acquisition and interpretation are too difficult in young children to make IONM useful [12]. More selective IONM use based on surgical complexity and patient risk, favoring targeted protocols over blanket application, is instead supported elsewhere [11].

These findings support selective, risk-adapted use of IONM in pediatric and adult spinal cord surgery, particularly for tethered cord and myelomeningocele repair, where it is associated with improved detection of neural compromise and better-documented outcomes in complex cases. This selective association does not establish that IONM is causally responsible for differences in outcomes, given the retrospective, non-randomized design of nearly all included studies. Universal use is not justified in lower-risk procedures such as FTL untethering. Broader implementation would benefit from larger cohorts, cost-effectiveness analysis, and standardized protocols that preserve flexibility for case-specific decisions.

Limitations and Future Directions

This review has several important limitations. Nearly all included studies were retrospective and observational, with no randomized comparisons; the association between IONM and neurological outcomes is therefore subject to confounding by indication, since patients selected for or against monitoring may have differed systematically in surgical complexity or preoperative risk. The 19 included studies also span a clinically heterogeneous range of diagnoses, including myelomeningocele, filum terminale lipoma, retethering after prior surgery, dermoid/epidermoid cysts, and other forms of spinal dysraphism, limiting direct comparability of outcomes. Reported IONM modalities, alert criteria, and anesthetic protocols varied substantially and were often incompletely described, and no formal risk-of-bias assessment was performed, consistent with the narrative rather than systematic nature of this review.

A further limitation concerns the search strategy: because this review was restricted to the PubMed database and to English-language publications, relevant studies indexed elsewhere or published in other languages may have been missed, and the sample may be affected by publication bias. Because the original search terms did not include tethered cord-specific vocabulary, a substantial share of the eligible literature required a second, expanded search branch to identify; although this was documented transparently in the PRISMA flow diagram, some eligible studies may still have been missed by keyword-based searching alone, as illustrated by one supplementary record identified only through manual citation tracking. The heterogeneity and quality of the included evidence did not support a reliable quantitative synthesis.

Two further directions are worth noting as hypothesis-generating rather than as priorities derived directly from this review. First, because several included studies focused on the immediate postoperative period, longitudinal studies following children well past adolescence could help clarify whether neurodevelopmental or psychiatric sequelae emerge later in life [1]. Second, given that infants born at lower-level birth hospitals tend to experience delayed repair with worse outcomes [23], remote neuromonitoring has been proposed as a potential way to extend IONM access to underserved or rural settings; this remains speculative, as the review did not identify any study directly evaluating remote IONM.

*Methodological Considerations and Clinical Utility*

A primary limitation of the synthesized literature is the predominance of Level IV evidence, consisting almost entirely of retrospective, single-center case series. This inherently elevates the risk of selection and recall bias across the pooled data. While the overall level of evidence is low, it represents the absolute highest tier of clinical evidence ethically and practically attainable in contemporary pediatric neurosurgery, where withholding IONM for a randomized control arm is widely considered unsafe. Consequently, this systematic synthesis holds immediate practical utility by mapping out known electrophysiologic-to-clinical outcome correlations and establishing standardized criteria to guide future prospective registries.

## Conclusions

IONM serves a defined role in the surgical management of spina bifida and complex spinal dysraphisms. Evidence from the 19 analyzed studies demonstrates that multimodal IONM incorporating MEP, SSEP, EMG, and BCR is clinically feasible and consistently associated with favorable postoperative motor outcomes. This clinical utility is most pronounced in high-risk scenarios, including adult presentations, reoperative detethering, and structurally complex reconstructions.

The current literature is heavily constrained by an overall moderate risk of bias, driven by a predominance of retrospective, single-center, non-comparative designs with unblinded outcome assessments. Methodological quality appraisal via the MINORS framework highlights a lack of prospective cohort tracking and consecutive patient recruitment. Consequently, available data establish clinical correlation rather than direct causation. Furthermore, the incremental value of IONM appears limited in low-risk procedures, such as isolated filum terminale lipoma release, where favorable outcomes are frequently achieved regardless of monitoring status.

To improve the quality of evidence, future research must shift away from isolated case series toward large-scale, multi-center prospective registries. Long-term follow-up protocols extending past the immediate postoperative window are essential to clarify the true durability of neurological, urological, and orthopedic outcomes. Ultimately, these efforts are required to establish standardized, risk-adapted protocols for the use of IONM in contemporary pediatric and adult neurosurgery.
